# Hydration for health hypothesis: a narrative review of supporting evidence

**DOI:** 10.1007/s00394-020-02296-z

**Published:** 2020-07-06

**Authors:** Erica T. Perrier, Lawrence E. Armstrong, Jeanne H. Bottin, William F. Clark, Alberto Dolci, Isabelle Guelinckx, Alison Iroz, Stavros A. Kavouras, Florian Lang, Harris R. Lieberman, Olle Melander, Clementine Morin, Isabelle Seksek, Jodi D. Stookey, Ivan Tack, Tiphaine Vanhaecke, Mariacristina Vecchio, François Péronnet

**Affiliations:** 1 Health, Hydration & Nutrition Science, Danone Research, Route Départementale 128, 91767 Palaiseau cedex, France; 2 Department of Kinesiology, University of Connecticut, Storrs, CT USA; 3 Hydration & Nutrition, LLC, Newport News, VA USA; 4 London Health Sciences Centre and Western University, London, ON Canada; 5 College of Health Solutions and Hydration Science Lab, Arizona State University, Phoenix, AZ USA; 6 Department of Physiology, Eberhard Karls University, Tübingen, Germany; 7 Westwood, MA USA; 8 Department of Clinical Sciences Malmö, Lund University, Malmö, Sweden; 9 Children’s Hospital Oakland Research Institute, Oakland, CA USA; 10 Explorations Fonctionnelles Physiologiques, Hôpital Rangueil, Toulouse, France; 11 École de Kinésiologie et des Sciences de l’activité Physique, Faculté de Médecine, Université de Montréal, Montréal, QC Canada

**Keywords:** Water, Renal, Metabolic, Arginine vasopressin, Copeptin

## Abstract

**Purpose:**

An increasing body of evidence suggests that excreting a generous volume of diluted urine is associated with short- and long-term beneficial health effects, especially for kidney and metabolic function. However, water intake and hydration remain under-investigated and optimal hydration is poorly and inconsistently defined. This review tests the hypothesis that optimal chronic water intake positively impacts various aspects of health and proposes an evidence-based definition of optimal hydration.

**Methods:**

Search strategy included PubMed and Google Scholar using relevant keywords for each health outcome, complemented by manual search of article reference lists and the expertise of relevant practitioners for each area studied.

**Results:**

The available literature suggest the effects of increased water intake on health may be direct, due to increased urine flow or urine dilution, or indirect, mediated by a reduction in osmotically -stimulated vasopressin (AVP). Urine flow affects the formation of kidney stones and recurrence of urinary tract infection, while increased circulating AVP is implicated in metabolic disease, chronic kidney disease, and autosomal dominant polycystic kidney disease.

**Conclusion:**

In order to ensure optimal hydration, it is proposed that optimal total water intake should approach 2.5 to 3.5 L day^−1^ to allow for the daily excretion of 2 to 3 L of dilute (< 500 mOsm kg^−1^) urine. Simple urinary markers of hydration such as urine color or void frequency may be used to monitor and adjust intake.

## Introduction

Water is the largest constituent of the human body, representing roughly 40 to 62% of body mass [1]. Water balance is constantly challenged by transepidermal, respiratory, fecal and urinary losses, with mean daily water turnover of 3.6 ± 1.2 L day^−1^ [2] or 2.8 to 3.3 and 3.4 to 3.8 L day^−1^ in women, and men, respectively [3]. Only a small amount of water is produced in the body (metabolic water, 0.25 to 0.35 L day^−1^ [2, 4]) and the human body has a limited capacity to store water; so water losses must be replaced daily. Thus, water has been called the ‘most essential’ nutrient [5, 6].

The maintenance of body water balance is so critical for survival that the volume of the body water pool is robustly defended within a narrow range, even with large variability in daily water intake. Evidence for this effective defense is found in population studies [4], observations of habitual low- vs. high-volume drinkers [7, 8], and water intake interventions [8–10], all of which demonstrate that large differences or changes in daily water intake do not appreciably alter plasma osmolality, thereby substantiating the stability of total body water volume. This tight regulation is governed by sensitive osmotic sensing mechanisms which trigger two key response elements: (1) the release of arginine vasopressin (AVP), which acts via vasopressin V2 receptors (V2R) on the renal collecting ducts, initiating renal water saving when water intake is low; (2) the triggering of the sensation of thirst to stimulate drinking.

Despite its importance, water is also referred to as a forgotten [11, 12], neglected, and under-researched [13] nutrient. This is reflected by discrepancies between regional water intake recommendations [4, 14], and the fact that these reference values represent Adequate Intakes (AIs). The AIs are based upon observed or experimentally derived estimates of average water intake with insufficient scientific evidence to establish a consumption target associated with a health risk or benefit. In practice, from the perspective of the general public, water may not even be visible in dietary guidelines (e.g., www.choosemyplate.gov). The implicit message is that there is little or no need to pay attention to water intake except in extreme situations; thirst is implicitly assumed to be an adequate guide.

### Hypothesis

This review advances the hypothesis that optimal water intake positively impacts various aspects of health. We propose an evidence-based definition of optimal hydration as a water intake sufficient to avoid excessive AVP secretion and to ensure a generous excretion of dilute urine, sufficient to avoid chronic or sustained renal water saving. For many, this would imply drinking somewhat beyond physiological thirst and likely more than the often-repeated target of ‘eight glasses of water per day’ called into question by Valtin [15] and others for lack of supporting evidence-based health outcomes. Here, we review the existing evidence for two specific mechanisms of action of how increased water intake may impact health: (1) the direct effect of increased urine flow on kidney and urinary tract health, and (2) the indirect effect of lowering AVP concentration on kidney and metabolic function. We conclude with a proposal for a range of water intake that provides optimal hydration.

### Literature review and search strategy

Searches for relevant literature were divided by subtopic. Each subtopic was investigated by a group of two to three authors and involved at least one expert with current, relevant clinical practice or recent research activity. Search strategy included PubMed and Google Scholar using relevant keywords for each health outcome (e.g., for kidney stones: kidney, stones, lithiasis, fluid, water, urine, flow, volume). This was accompanied by manual search of article reference lists and the publication knowledge and expertise of relevant practitioners for each area studied (e.g., nephrology, physiology, metabolic health). For health outcomes included in Tables 1 and 2, only human studies (observational or interventional) were included; animal or mechanistic work is cited where relevant to describe or support a plausible mechanism. No systematic assessment of study quality was performed. The initial search included articles available through the end of 2018; however, subsequent modifications to the manuscript resulted in the inclusion of some more recent references.

**Table 1:** Studies reporting a relationship between fluid intake and/or urinary hydration biomarkers and health outcomes related to urine dilution: kidney stones and urinary tract infection

Author (year) Study type, cohort name, follow-up period Population	Fluid intake or urinary hydration marker associated with health outcome		Health Outcome (Risk or *Benefit*)
	Total fluid intake volume (TFI, L·day^−1^)	24-h urine volume (UVol, L·day^−1^) or Urine osmolality (UOsm, mOsm·kg^−1^)	
Borghi et al. (1996) [23] Case–control Recurrent stone formers vs. healthy controls		UVol, *Mean [sd]* Stone formers: 1.04 [0.24] Controls: 1.35 [0.53]	Risk: Stone formers had lower spontaneous 24 h urine volume than age, sex, body weight, and socioeconomic-matched controls
Borghi et al. (1996) [23] RCT, 5-year follow-up Recurrent stone formers		UVol, *Mean [sd]* Intervention: Pre: 1.1 [0.2] Post: 2.6 [0.4] Control: Pre: 1.0 [0.2] Post: 1.0 [0.2]	*Benefit:* Increasing urine volume reduced kidney stone recurrence (12% vs. 27% in control group), time between episodes, and urine supersaturation in stone formers
Curhan et al. (2004) [117] Prospective, NHS II cohort, 8-year follow-up General population (women)	TFI, *quintiles* Q1: ≤ 1.43 Q2: 1.43–1.85 Q3: 1.85–2.25 Q4: 2.25–2.77 Q5: ≥ 2.77		*Benefit:* Reduction in multivariate-adjusted RR for incident kidney stones in women in Q3, Q4, and Q5 (RR 0.79, 0.72, and 0.68, respectively), compared to reference (women with FI ≤ 1.43 L·day^−1^)
Curhan & Taylor (2008) [24] Pooled retrospective study of 3 cohorts (NHS I, NHS II, HPFS) General population		UVol, *Cutoff value* From 1.5 to ≥ 2.5	*Benefit:* Across three cohorts including 2,237 stone formers, individuals with a urine volume ranging from 1.5L to more than 2.5L·day^−1^ were shown to be at lower risk of developing kidney stones with corresponding RR ranging from 0.46 (urine volume 1.5 to 1.74L·day^−1^) to 0.22 (urine volume ≥ 2,5 L·day^−1^), compared to reference (urine volume ≤ 1.0L·day^−1^)
Curhan et al. (1993) [22] Prospective cohort (HPFS), 4-year follow up General population (men)	TFI, *quintiles* Q1: < 1.28 Q2: 1.28–1.67 Q3: 1.67–2.05 Q4: 2.05–2.54 Q5: ≥ 2.54		*Benefit:* Reduction in multivariate-adjusted RR for incident kidney stones in men in Q5 (RR = 0.71), compared to reference (men with FI < 1.28 L·day^−1^)
Hooton et al. (2018) [42] RCT, 12-month follow-up Recurrent UTI (women)	TFI (intervention group), *Mean [sd]* Pre: 1.1 [0.1] Post: 2.8 [0.2]	UVol (intervention group), *Mean [sd]* Pre: 0.9 [0.2] Post: 2.2 [0.3] UOsm (intervention group), *Mean [sd]* Pre: 721 [169] Post: 329 [117]	*Benefit:* 48% reduction in UTI recurrence in intervention group vs. control; increased time between episodes; reduction in antibiotic use

Empty cells denote that this variable was not reported

*HPFS* Health Professionals Follow-Up Study; *NHS I* Nurses’ Health Study; *NHS II* Nurses’ Health Study II; *RCT* Randomized Controlled Trial; *RR* Relative risk; *sd* Standard Deviation; *TFI* Total Fluid Intake, volume of drinking water plus other beverages; *UTI* Urinary Tract Infection

## Direct effect of increased water intake to increase urine flow

While total body water and plasma osmolality are defended within a narrow range, urine volume adjusts water losses to compensate for fluctuations in daily water intake and insensible losses. Urine output adjusts quickly to changes in water intake, and 24-h urine volume is a reasonable surrogate marker for high or low daily water intake in healthy adults in free-living conditions [16]. Here, we review the evidence for the importance of high urine flow in the secondary prevention of kidney stones and urinary tract infection. A detailed description of individual studies is provided in Table 1.

### Kidney stones

Kidney stones are hard crystalline mineral deposits that form inside the kidney or urinary tract. They occur in 10% of the population worldwide [17] and recurrence is high: 40 to 60% of stone formers will relapse within 5 years following a first episode [18–20]. Stone formation results from dietary, genetic and/or environmental factors. In particular, low fluid intake and low urine volume have been shown to be significant risk factors for kidney stones in first-time and recurrent stone formers (Table 1) [21–24]. Mechanistically, low urine volume leads to higher concentrations of urinary solutes and promotes urine supersaturation, favoring crystal nucleation and stone growth [25]. Conversely, increased water intake facilitates the flushing of crystals by increasing urine flow.

In a 5-year randomized controlled trial (RCT), patients were either instructed to increase water intake to achieve a urine volume of 2 L day^−1^ without any further dietary changes or were assigned to a control group receiving no intervention [23]. Over the follow-up period, the recurrence of stones was lower (12%) in the intervention group, who maintained a urine volume of more than 2.5 L day^−1^, compared with the control group (27% recurrence) whose urine volume remained at about 1.2 L day^−1^. Two systematic reviews on this topic have concluded that high water intake reduces long-term risk of kidney stone recurrence [26, 27]. In agreement with these findings, the European Association of Urology and the American Urological Association current guidelines for the secondary prevention of kidney stones recommend stone-formers maintain a fluid intake that will achieve a urine volume of at least 2.0 to 2.5 L daily [28, 29]. Interestingly, increasing fluid intake also appears to be perceived as one of the easiest lifestyle changes to make with respect to stone recurrence. While dietary factors also influence stone formation, patients with recurrent kidney stones reported being more confident in their ability to increase fluid intake, compared to changing other dietary factors or taking medicine [30].

In terms of primary prevention, we are only aware of one study investigating the effects of increased habitual fluid intake [31]. In an area of Israel with a high incidence of urolithiasis, healthy inhabitants of one town participated in an education program that encouraged adequate fluid intake, while inhabitants of a second town did not participate in the program. At the end of the 3-year study period, urine output was found to be higher and incidence of urolithiasis lower in the intervention group compared with the control group. To date, no recommendation for primary stone prevention has been proposed. However, considering the aggregate of observational evidence, including a successful RCT for secondary prevention, as well as a clear mechanism of urine dilution to avoid supersaturation and stone formation, increased water intake among low drinkers in general would appear to be a reasonable, easy and cost-effective way to reduce urolithiasis recurrence in known stone formers [32] as well as in primary prevention [33].

### Urinary tract infection

Urinary tract infections (UTI) are bacterial contaminations of the genitourinary tract affecting a large part of the female population and resulting in general discomfort and decreased quality of life. Increased water intake is sometimes recommended in clinical practice as a preventive strategy for UTI in women suffering recurrent events. However, the empirical evidence for any relationship between UTI and water intake or urinary markers of hydration is equivocal. Several non-randomized studies reported that low intake of fluids or reduced number of daily voids are associated with increased risk of UTI [34–38]. In contrast, other published data show no association between fluid intake and the risk of UTI, no difference in fluid intake between women with recurrent infections and healthy controls, and no effect of increased water intake on UTI risk [39, 40]. A small crossover trial published in 1995 demonstrated that self-assessment of urine concentration encouraged lower urine osmolality and reduced frequency of UTI [41]; however, the study had a number of methodological problems including large number of participants lost to follow-up, lack of a proper control group, and not reporting fluid intake.

Recently, Hooton et al. published the first RCT assessing the effect of increased water intake on the frequency of acute uncomplicated lower UTI in premenopausal women [42]. One hundred and forty women suffering from recurrent UTI with low fluid intake and low urine volume were randomly assigned to increase their daily water intake by 1.5 L or to maintain their usual intake for 12 months. Increasing water intake (to 2.8 L day^−1^) and urine volume (to 2.2 L day^−1^) resulted in a 48% reduction in UTI events. Of note, a second benefit to increasing water intake was a reduction of antibiotic use, for prophylaxis or treatment of UTI. The proposed mechanism for the improvement in UTI recurrence was that increasing void frequency and urine volume facilitated the flushing of bacteria and thus reduced bacterial concentration in the urinary tract. More recently, a second study of elderly patients in residential care homes found that encouraging increased fluid intake by implementing structured ‘drink rounds’ multiple times per day reduced UTIs requiring antibiotics by 58%, and UTIs requiring hospital admission by 36% [43]. While the study did not measure individual increases in fluid intake during the intervention, the magnitude of reduction in UTI is substantial, and similar to that reported by Hooton et al. in a younger population, supporting the role for increased fluid intake in the secondary prevention of UTI.

### Take home points

- Increasing fluid intake is effective in the secondary prevention of kidney stones and urinary tract infection. Little is known about whether high fluid intake is also effective in primary prevention.
- Mechanistically, increasing fluid intake results in lower urine concentration and increased urine flow. The former may be important in preventing supersaturation and crystal formation, while the latter encourages frequent flushing of the urinary tract which may be helpful for both kidney stone and UTI prevention.
- European and American urological associations encourage maintaining a fluid intake sufficient to produce 2 to 2.5 L of urine per day to reduce risk of stone formation.

## Indirect effect of increased water intake: mechanisms mediated by reducing circulating AVP

AVP is a critical hormone for the regulation of body fluid homeostasis. It can be secreted in response to small fluctuations of serum osmolality and primarily regulates fluid volume through its antidiuretic action on the kidney. Binding of AVP to the V2-receptors (V2R) located in the renal collecting ducts, induces translocation of aquaporin-2 to the cellular membrane and allowing increased water reabsorption [44] and the defense of total body water and plasma osmolality. Copeptin, a stable C-terminal fragment of the AVP precursor hormone released in a 1:1 ratio with AVP, is a surrogate marker for AVP secretion [45]. The recent availability of an ultra-sensitive assay for copeptin has dramatically increased research on AVP or copeptin and health outcomes. Lower circulating copeptin is associated with improved metabolic and renal outcomes (Table 2).

**Table 2:** Studies reporting a relationship between between fluid intake, urinary hydration markers, and/or copeptin, and health outcomes related to metabolic disease and kidney decline

Author (year) Study type, cohort name, follow-up period Population	Fluid intake, urinary hydration biomarker or copeptin value associated with health outcome			Health Outcome (Risk or *Benefit*)	
	Total water intake, total fluid intake, or plain water intake (L·day^−1^)	24-h urine volume (UVol, L·day^−1^) or concentration (UOsm, mOsm·kg^−1^ or USG, unitless)	Plasma copeptin (pmol·L^−1^)		
Abbasi et al. (2012) [57] Prospective, PREVEND cohort, 7.7-year follow-up General population			*Median [25,75th percentile]* Men Q4: 12.5 [10.5,15.5] Women Q3: 4.4 [4.0,4.9] Q4: 7.6 [6.3,9.8]	Risk: Increased multivariate-adjusted odds of incident T2D in women, but not men, during 7.7 year follow up, starting from the third quartile of baseline copeptin compared to Q1 (reference: 1.8 [1.4–2.1] pmol·L^−1^) Risk: Increased odds of incident T2DM in men (more marginally significant, depending on adjustment model) in the highest quartile of copeptin compared to Q1, 3.0 [2.3–3.5] pmol·L^−1^	
Boertien et al. (2013) [118] Prospective, ZODIAC-33, 6-year follow up T2DM			*Quintiles* Q1 < 3.11 Q5 > 8.96	Risk: Highest quartile of copeptin had the most rapid rate of eGFR decline and largest change in albumin:creatinine ratio compared to reference (Q1)	
Clark et al. (2011) [79] Prospective, Walkerton Health Study Cohort, 6-year follow up General population		UVol, *quartiles* < 1.0 1–1.9 2–2.9 ≥ 3.0		Risk: urine volume (< 1.0 L·day^−1^) more likely to demonstrate mild to moderate kidney function decline, compared to reference (1–1.9 L·day^−1^), odds ratio multivariate adjusted *Benefit:* High urine volume ≥ 3 L·day^−1^ less likely to demonstrate mild to moderate or severe kidney function decline, compared to reference (1–1.9 L·day^−1^), odds ratios multivariate adjusted	
El Boustany et al. (2018) [54] Prospective, pooled analysis of 3 cohorts: DESIR, MDC-CC, PREVEND, 8.5–16.5-year follow-up General population			*Tertiles, Median [IQR]* Men T2: 5.9 [1.7] T3: 10.6 [4.6] Women: T2: 3.5 [1.0] T3: 6.5 [3.1]	Risk: Top tertile (T3): Higher fasting plasma glucose and triglycerides compared to T2, T1; Risk: Top two tertiles (T2 and T3): kidney function decline compared to T1 Reference copeptin T1 3.2 [1.4], 2.1 [0.8] pmol·L^−1^ for men and women, respectively	
Enhorning et al. (2010) [55] Prospective, MDC-CC, 12-year follow-up General population			*Median [25,75th percentile]* 6.74 [4.44,10.9]	Risk: Baseline copeptin in participants developing T2DM during 12-year follow-up; compared to 4.90 [3.03–7.65] pmol·L^−1^ in those not developing T2DM (all participants NFG at baseline)	
Enhorning et al. (2011) [52] Cross-sectional, MDC-CC General population			Men: ≥ 10.7 or Women: ≥ 6.47	Risk: Higher likelihood of hypertension, high CRP or abdominal obesity, multivariate-adjusted	
Enhorning et al. (2011) [52] Cross-sectional, MDC-CC General population			Men: ≥ 4.61 or Women: ≥ 2.72	Risk: Higher likelihood of Metabolic Syndrome (age and sex adjusted only)	
Enhorning et al. (2013) [119] Prospective, MDC-CC, 15.8-year follow up General population			*Median [25,75th percentile]* Men Q3: 8.44 [7.64,9.53] Q4: 13.5 [11.5,16.6] Women Q3: 5.14 [4.70,5.76] Q4: 8.41 [7.22,10.45]	Risk: Third and fourth quartiles of copeptin: more likely to develop abdominal obesity, T2DM (age- and sex-adjusted) Risk: Fourth quartile of copeptin: More likely to develop metabolic syndrome (age and sex adjusted); abdominal obesity, microalbuminuria (multivariate adjusted)	
Enhorning et al. (2018) [66] RCT, 6-week follow up General population	TWI, *Median [25,75th percentile]* Pre: 1.9 [1.6,2.1] Post: 2.7 [2.3,3.1]	UVol, *Median [25,75th percentile]* Pre: 1.06 [0.9,1.2] Post: 2.27 [1.52,2.67] UOsm*, Median [25,75th percentile]* Pre: 879 [705,996] Post: 384 [319,502]	*Median [25,75th percentile]* Pre: 12.9 [7.4,21.9] Post: 7.8 [4.6, 11.3]	*Benefit*: Increasing plain water intake by 0.9 L·day^−1^ resulted in a reduction in copeptin and was accompanied by a lowering of fasting plasma glucose: from *(mean [sd]* 5.94 [0.44] to 5.74 [0.51] mmol·L^−1^ over a 6-week follow-up	
Meijer et al. (2010) [120] Cross-sectional, PREVEND General population		UVol, *Mean by quintile of copeptin (Q2-Q4 estimated from figure)* Men Q1: 1.74 Q2: 1.60 Q3: 1.55 Q4: 1.45 Q5: 1.36 Women Q1: 1.82 Q2: 1.70 Q3: 1.60 Q4: 1.55 Q5: 1.43	*Quintiles:* Men Q1: 0.6–3.7 Q2: 3.8–5.3 Q3: 5.4–7.3 Q4: 7.4–10.5 Q5: 10.5–632 Women Q1: 0.1–2.1 Q2: 2.2–3.0 Q3: 3.1–4.2 Q4: 4.3–6.2 Q5: 6.3–131		Risk: Higher copeptin was associated with higher urinary albumin excretion, greater prevalence of microalbuminuria, low urine volume and high urine osmolality in men and women, multivariate-adjusted. Higher copeptin also significantly associated with lower eGFR (men and women), and in men only, higher plasma glucose, prevalent T2DM, higher serum CRP, and higher serum creatinine
Roussel et al. (2016) [56] Prospective, DESIR cohort, 9-year follow-up General population			*Quartiles* Q1: 0.91–2.92 Q2: 2.93–4.05 Q3: 4.06–6.57 Q4: 6.58–115		*Benefit*: Cumulated incident IFG or T2DM by quartile: 11, 14.5, 17.0, 23.5%, respectively in men and women
Sontrop et al. (2013) [77] Cross-sectional, NHANES General population	Plain water intake, *Median of bottom 20%* 0.5 L·day^−1^				Risk: More likely to have moderate CKD (multivariate-adjusted OR), compared to those with high plain water intake (top 20%; median 2.6 L·day^−1^)
Strippoli et al. (2011) [76] Cross-sectional, Blue Mountains, Australia General population	TFI, *median of quintile* Q1: 1.8 Q5: 3.2				*Benefit*: Reduced risk of moderate CKD in the highest quintile compared to reference (Q1)
Velho et al. (2018) [121] Prospective, pooled DIABHYCAR and SURDIAGENE cohorts, 4.7-year follow up T2DM			*Tertiles* *median [IQR]* T3: 13.5 [6.5] and 16.2 [12.2] in both cohorts, respectively		Risk: Highest tertile of copeptin had increased (multivariate-adjusted) risk of MI, coronary revascularization, CHF, cardiovascular events, cardiovascular death, rapid kidney function decline, doubling of serum creatinine or ESRD, over a median 4.7-year follow up, compared to the lowest copeptin tertile (reference 3.7 [2.0] and 3.1 [1.9] pmol·L^−1^, respectively)
Wennamethee et al. (2015) [58] Prospective, BRHS, 13-year follow-up General population (men 60–79 years)			*Quintiles* Q1: < 2.18 Q2: 2.18–3.12 Q3: 3.13–4.45 Q4: 4.46–6.78 Q5: ≥ 6.79		Risk: Higher incident T2DM in Q5 versus all other quintiles, multivariate-adjusted

Empty cells denote that this variable was not reported

*BRHS* British Regional Heart Study; *CRP* C-reactive protein; *DESIR* Devenir des Spondylarthropathies Indifférenciées Récentes Cohort; *DIABHYCAR* Non-Insulin-Dependent Diabetes, Hypertension, Microalbuminuria or Proteinuria, Cardiovascular Events, and Ramipril Trial; *MDC-CC* Malmö Diet and Cancer Study, Cardiovascular Cohort; *NFG* Normal fasting glucose; *NHANES* National Health and Nutrition Examination Survey; *PREVEND* Prevention of Renal and Vascular End-stage Disease Cohort; *RCT* Randomized controlled trial; *RR* Relative risk; *SURDIAGENE* Survival Diabetes and Genetics Cohort; *T2DM* Type 2 diabetes mellitus; *TFI* Total Fluid Intake, volume of drinking water plus other beverages; *TWI* Total Water Intake, water coming from fluids and food; *ZODIAC-33* Zwolle Outpatient Diabetes project Integrating Available Care Cohort

### AVP and metabolic dysfunction

In addition to its well-defined role in concentrating urine and regulating body water via the V2R, AVP also acts on other AVP receptors (V1aR and V1bR) which occur in a variety of central and peripheral tissues, with multiple and wide-ranging physiological effects [46]. AVP may play an important role in the development of metabolic disease because it stimulates hepatic gluconeogenesis and glycogenolysis through V1aR [47, 48] and triggers release of both glucagon and insulin through V1bR in pancreatic islets [49]. Moreover, AVP stimulates the release of adrenocorticotrophic hormone (ACTH) via V1bR in the anterior pituitary gland, thereby leading to elevated adrenal cortisol secretion and prompting undesirable cortisol-mediated gluconeogenesis [50, 51].

High plasma copeptin levels have been associated with insulin resistance and metabolic syndrome in cross-sectional population and community-based studies [52, 53]. Pooled data from three large European cohorts also show that participants in the top tertile of copeptin have higher fasting plasma glucose compared to the bottom and medium tertiles, and are more likely to have type 2 diabetes (T2DM) [54]. Moreover, copeptin has been consistently identified as an independent predictor of T2DM in four European cohorts (Table 2) [55–58], suggesting that AVP contributes to the development of the disease. Furthermore, within diabetic patients, individuals with the highest copeptin level had higher HbA1c levels [59], were more likely to develop metabolic complications, heart disease, death and all-cause mortality [60, 61].

A causal role for AVP in metabolic disorders is supported by preclinical evidence showing that high AVP concentration impairs glucose regulation in rats, an effect reversed by treatment with a selective V1aR antagonist [62, 63]. In humans, causality is also supported by recent evidence from a Mendelian randomization approach study which reported that certain single nucleotide polymorphisms within the AVP-neurophysin II gene were associated with both higher AVP and higher incidence of impaired fasting glucose in men, but not in women [56].

Individuals with lower habitual fluid intake have higher AVP levels compared to those who consume more fluids, despite similar plasma osmolality [7, 64], and increasing plain water intake can lower AVP or copeptin over hours, days, or weeks [10, 64, 65]. Compellingly, the most substantial reductions in copeptin appear to occur in those with insufficient water intake as indicated by high baseline urine osmolality, low urine volume and/or higher baseline copeptin level [65, 66]. Epidemiological evidence is inconsistent: low water intake is linked with increased risk of new-onset hyperglycemia [67], and an association between plain water intake and elevated glycated hemoglobin has been noted in men, but not women [68]. Pan et al. also found no association between plain water intake and incident T2DM in a large cohort of women [69]. In the short-term, a six-week pilot study in adults with high urine osmolality, low urine volume, and high copeptin, demonstrated that increasing water intake reduced circulating copeptin and resulted in a small but significant reduction in fasting plasma glucose, but no changes in fasting plasma insulin or glucagon [66]. However, a recent perspective paper pointed out that different manipulations to hydration have produced inconsistent results, suggesting that the relationship between water intake, hydration, AVP and metabolic response may be more complex [70].

Overall, there is convergent epidemiological evidence and a plausible mechanism for how higher circulating AVP may contribute to increased risk for metabolic disease. There is also evidence from short-term studies that in individuals with higher AVP, increasing water intake can have an AVP-lowering effect [10, 64, 65]. However, longer-term studies are needed to demonstrate whether lowering AVP through increased water intake is effective in maintaining metabolic health.

### Lower AVP and renal water saving in chronic kidney disease (CKD)

The rationale for use of water as a treatment in CKD is based on its ability to suppress the secretion and thus the detrimental effects of AVP on the kidneys [71, 72]. AVP increases renal hyperfiltration and renal plasma flow with its associated proteinuria, hypertension and renal scarring [73, 74]. AVP antagonists reduce proteinuria, lower blood pressure and prevent renal injury. Water intake acts as an AVP antagonist, as shown by the experimental animal work of Bouby and Bankir in 1990 which demonstrated the therapeutic role of increased hydration in slowing progressive loss of kidney function [72].

Water intake and its relationship with AVP in patients with CKD is documented by various human observational studies assessing hydration as a potential therapy in CKD. However, there are inconsistencies in these studies regarding the possible benefits of increased water intake to slow and prevent CKD [75–79]. Briefly, cross-sectional studies in Australian and American cohorts have reported a kidney protective effect of higher fluid intake [76] and lower prevalence of CKD in participants reporting higher plain water intake, a beneficial effect not observed for any other type of beverage [77]. In contrast, a second prospective study analyzing longitudinal data of the same Australian cohort reported no significant association between total fluid intake and longitudinal loss of kidney function [78]. This apparent contradiction with the previous analysis may be due to the fact that plain water intake, a major driver of high fluid intake [80], was excluded from analysis. Finally, a 7-year longitudinal study of over 2000 Canadians that controlled for multiple baseline variables also demonstrated that higher urine volumes significantly predicted slower renal decline [79]. These observations are further strengthened by a longitudinal study of more than 2000 CKD patients with 15-year median follow-up demonstrating that those in the highest quartile of fluid intake had better survival outcomes than those in the lowest quartile [81].

To our knowledge there exists a single RCT on water intake in CKD prevention. In a six-week pilot study of 29 patients with stage 3 CKD, Clark et al. showed that an increased urine volume of 0.9 L was associated with a significant reduction in copeptin without any toxicity or measurable change in quality of life [82, 83]. This pilot study led to the Water Intake Trial [84], a parallel-group RCT in which adults with stage 3 CKD and microalbuminuria were either coached to increase water intake by 1 to 1.5 L day^−1^ above their usual intake (high water intake (HWI) group), or to maintain usual water intake. The primary analysis at 1-year follow-up demonstrated that a 0.6-L increase in urine output in the HWI group versus the control group was associated with a small but significant reduction in copeptin, but not associated with a difference in albuminuria nor in estimated glomerular filtration rate (eGFR). However, this trial may have focused on the wrong population, as the majority of participants ingested approximately 2–3 L of fluid per day at baseline; consequently, the margin for improved hydration was small. Future RCTs should consider focusing on the role of increased hydration in low water drinkers with high copeptin levels and thus higher potential to respond to increased water intake, include more precise measures of renal function and possibly a longer follow-up.

### Autosomal dominant polycystic kidney disease

Autosomal dominant polycystic kidney disease (ADPKD) is a genetic disorder characterized by development and enlargement of multiple cysts in the kidney, leading to loss of renal function, hypertension, and renal failure in 50% of patients by the age of 60 [85]. The major sites of cyst development in ADPKD are the collecting ducts and distal nephrons, where cyclic adenosine monophosphate (cAMP) stimulates both epithelial cell proliferation and fluid secretion [86]. Since AVP is a strong activator of cAMP in these *loci* [87, 88], the rate of progression of the disease is associated with its circulating concentration: a loss of urinary concentrating ability early in ADPKD is associated with a concomitant rise in AVP [89–93]. Further, preclinical studies demonstrate that ADPKD progression is slower in animals lacking AVP, and that in AVP knock-out animal models, desmopressin, a synthetic AVP analogue, accelerates disease progression [87, 94].

Reducing AVP action represents a recent therapeutic target for patients with ADPKD, with two possible mechanisms: (1) blocking its receptors; more specifically the V2R in the collecting ducts; or (2) decreasing circulating AVP. Administration of vaptans, a class of nonpeptide AVP receptor antagonists, in particular tolvaptan, an oral selective antagonist of the V2R, decrease cAMP in epithelial cells of the collecting ducts and distal nephron [95]. A recent RCT reported inhibition of the action of AVP by tolvaptan significantly slows the rate of disease progression [96].

The suppression of AVP by increasing water intake could also slow renal cyst growth in ADPKD [87, 88, 94, 96, 97]. Rodent models of polycystic kidney disease have shown AVP suppression by increased water intake is associated with a significant renal-protective effect [87]. However, data available in humans are limited and conflicting. A positive effect of high water intake on ADPKD was observed in one post hoc analysis [98] and two short-term interventional trials [98, 99] while a negative effect of high water intake was reported in a small observational cohort study [100] One large RCT is currently underway to determine the efficacy and safety of increasing water intake to prevent the progression of ADPKD over a 3-year period [101].

### Take home points

- AVP, or the antidiuretic hormone, is most well-known for its central role in maintaining body water balance. However, AVP can also stimulate hepatic gluconeogenesis and glycogenolysis and can moderate glucose-regulating and corticotrophic hormones through its V1a and V1b receptors. The AVP-V2 receptor is also implicated in the pathophysiology of a particular form of kidney disease (ADPKD).
- In epidemiological studies, higher circulating AVP, measured by its equimolar surrogate, copeptin, is associated both cross-sectionally and longitudinally with higher odds for kidney function decline, components of the metabolic syndrome, and incident T2DM.
- Short-term intervention studies suggest that in individuals with higher AVP, increasing water intake can have an AVP-lowering effect. However, it is unclear whether lowering AVP through increased water intake will reduce disease risk.

## Optimal hydration

If water intake may contribute to maintaining kidney and metabolic health, what would constitute optimal hydration and how much water should one consume?

Based on the evidence above, optimal hydration should result in excretion of a generous volume of dilute urine, sufficient to avoid chronic or sustained renal water saving and excess AVP secretion. Individual needs vary; nonetheless, the available data (Tables 1, 2) provide a starting point for practical and evidence-based recommendations.

The first recommendation is that beyond replacing daily fluid losses, optimal hydration should be viewed as allowing the excretion of a sufficient urine volume to avoid urine concentration and supersaturation. Based on the evidence for fluid intake, urine volume, and kidney stones and UTI, it would appear reasonable to maintain a volume of excreted urine of 2 to 3 L per day. To account for other avenues of water loss (insensible, fecal [4, 14]), achieving a urine volume of 2 to 3 L would require consuming a fluid volume slightly higher than the AIs currently proposed by EFSA [14], and approaching the IOM AIs [4]. We suggest that *daily total water intake* for *healthy adults* in a *temperate climate*, performing, at most, *mild to moderate physical activity* should be 2.5 to 3.5 L day^−1^. While total water intake includes water from both food and fluids, plain water is the only fluid the body needs. Plain water and other healthy beverages should make up the bulk of daily intake. A practical, evidence-based scoring tool for evaluating healthy beverage choices has been proposed by Duffey et al. [102].

**Fig. 1:**
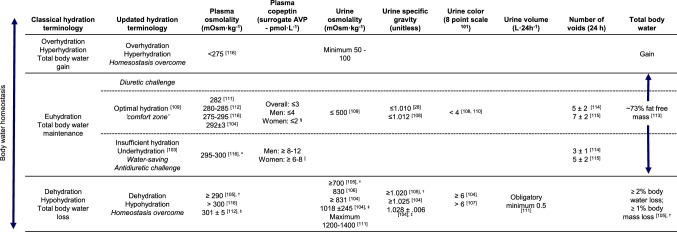
Terminology and associated cut-off values for common biomarkers of hydration.^*^Defined as ‘impending dehydration’. ^†^In the original text, these values are described as limits for euhydration (e.g., POsm < 290, UOsm < 700). For clarity we have positioned these values as limits for dehydration (e.g., POsm ≥ 290, UOsm ≥ 700) in order to avoid the interpretation that these values were limits for insufficient hydration. ^‡^Decision level for 95% probability of dehydration. ^§^Approximate range of plasma copeptin in bottom quartile or other reference interval (lowest risk for kidney or cardiometabolic disease)—see Table 2. ^||^Approximate range of plasma copeptin for increased risk for kidney or cardiometabolic disease—see Table 2

The second recommendation, for healthy individuals as well as in those with metabolic dysfunction, is to drink enough to reduce excessive AVP secretion as this may be beneficial for the kidney and reduce metabolic risk. This is especially relevant for individuals who may be underhydrated [103], with low 24 h urine volume or high urine concentration suggestive of AVP secretion linked to insufficient water intake. While higher circulating AVP is associated with increased disease risk, to date there is insufficient data to suggest a level of copeptin which may be appropriate to target for risk reduction. However, the use of urinary biomarkers of hydration such as osmolality can provide useful information reflecting urine concentrating and diluting mechanisms and overall antidiuretic activity. Multiple authors have proposed cut-offs representing de- or hypohydration for several urinary and plasma biomarkers (Fig. 1), conversely, suggestions for optimal hydration are infrequently provided [28, 104–116]. Several years ago a cutoff of 500 mOsm kg^−1^ was proposed as a reasonable target for optimal hydration, based on retrospective analyses of existing data [109] indicating that this cut-off would represent sufficient water intake to produce adequate urine volume with respect to kidney health risk, and reduce antidiuretic effort and circulating AVP. Today, several RCTs have demonstrated that lowering 24 h urine osmolality to approach 500 mOsm kg^−1^ or below can reduce circulating copeptin [10, 64, 65] as well as improve metabolic markers [66] and reduce UTI incidence [42]. For clinician or home use, maintaining a urine specific gravity of less than 1.013, or a urine color of 3 or below [108] on an eight-point color scale [107], or a 24 h void frequency of at least 5 to 7 voids daily [114, 115] are suggestive of a fluid intake sufficient to achieve optimal hydration (Fig. 1). As color and void frequency are accessible without specific laboratory instruments, they may be used by the general population for daily hydration awareness.
